# Translation and validation of the Hungarian Version of the infection control standardized questionnaire: a cross-sectional study

**DOI:** 10.1186/s12912-022-01024-8

**Published:** 2022-09-02

**Authors:** Sahar Hammoud, Faten Amer, Haitham Khatatbeh, Huda Alfatafta, Miklós Zrínyi, Béla Kocsis

**Affiliations:** 1 Doctoral School of Health Sciences, Faculty of Health Sciences, University of Pécs, Vörösmarty u. 4, 7621 Pécs, Hungary; 2 Basic Health Sciences and Health Visiting, Faculty of Health Sciences, Institute of Nursing Sciences, University of Pécs, Vörösmarty u. 4, H-7621 Pécs, Hungary; 3 Department of Medical Microbiology and Immunology, Medical School, University of Pécs, Szigeti u. 12, 7624 Pécs, Hungary

**Keywords:** Factor analysis, statistical, Health knowledge, attitudes, practice, Infection control, Nurses, Reliability, Validation studies as topic

## Abstract

**Background:**

To our knowledge, there is currently no psychometrically validated Hungarian scale to evaluate nurses’ knowledge about infection prevention and control (IPC) practices. Thus, we aim in this study to assess the validity and reliability of the infection control standardized questionnaire Hungarian version (ICSQ-H).

**Methods:**

A cross-sectional, multisite study was conducted among 591 nurses in Hungary. The original ICSQ included 25 questions. First, the questionnaire was translated into Hungarian. Then, content validity was assessed by a committee of four specialists. This was done by calculating the item content validity index and scale content validity index. Afterward, structural validity was evaluated in a two-step process using principal component analysis and confirmatory factor analysis. The goodness of fit for the model was measured through fit indices. Convergent validity was assessed by calculating the average variance extracted. Additionally, discriminant validity was evaluated by computing the Spearman correlation coefficient between the factors. Finally, the interitem correlations, the corrected item-total correlations, and the internal consistency were calculated.

**Results:**

The content validity of the questionnaire was established with 23 items. The final four-factor ICSQ-H including 10 items showed a good fit model. Convergent validity was met except for the alcohol-based hand rub (ABHR) factor, while discriminant validity was met for all factors. The interitem correlations and the corrected item-total correlations were met for all factors, but the internal consistency of ABHR was unsatisfactory due to the low number of items.

**Conclusions:**

The results did not support the original three-factor structure of the ICSQ. However, the four-factor ICSQ-H demonstrated an adequate degree of good fit and was found to be reliable. Based on our findings, we believe that the ICSQ-H could pave the way for more research regarding nurses’ IPC knowledge to be conducted in Hungary. Nevertheless, its validation among other healthcare workers is important to tailor effective interventions to enhance knowledge and awareness.

**Supplementary Information:**

The online version contains supplementary material available at 10.1186/s12912-022-01024-8.

## Background

Infection prevention and control (IPC) is one of the most cost-effective interventions to prevent the transmission of healthcare-associated infections (HAIs) [1] and disease outbreaks and to ensure the safety of healthcare workers (HCWs) [2]. The proper implementation of IPC measures may result in a 70% reduction in HAIs [3]. IPC practices have been present in different forms for decades. Universal precautions (UPs) were first introduced by the Centers for Disease Control and Prevention in the early 1980s after the identification of acquired immunodeficiency syndrome as a means of ensuring HCW safety. In 1996, UPs were replaced by standard precautions (SPs) after being revised. Later, IPC guidelines were updated several times as a result of several disease outbreaks [4]. For instance, respiratory hygiene/cough etiquette was added after the emergence of the severe acute respiratory syndrome epidemic in 2003. Furthermore, safe injection practices were included after the continued outbreaks of hepatitis B and C [4]. Afterward, the guidelines were further updated after the 2014 Ebola virus disease outbreak in West Africa [5].

Implementing IPC measures is a mandatory requirement in all healthcare institutions, yet despite policies and procedures to impose their practice, HCWs’ compliance with IPC remains substandard [4]. Poor knowledge of IPC is the main reason for the low adherence of HCWs to IPC practices. Other common reasons are organizational barriers, insufficient supplies, time limits, poor experience, inadequate training, and poor self-efficacy [4, 6–9]. Attempts should be continued to enhance the knowledge of HCWs on IPC to ensure higher compliance with IPC practices. Efforts should focus on nurses, who play a vital role in controlling and preventing the transmission of HAIs [9], which have detrimental effects on patient safety [10].

A recent systematic review on nurses’ knowledge and practice of IPC measures reported a lack of investigation of the validity and reliability in most of the included studies [11]. Given this premise, a valid and reliable tool is required to assess nurses’ knowledge about IPC measures. The infection control standardized questionnaire (ICSQ) is an instrument that was developed by Tavolacci et al. [12] to measure IPC knowledge among HCWs, including nurses. The ICSQ assesses knowledge about SPs, including their indications, and the use of personal protective equipment (PPE) (gloves, masks, gowns), as well as knowledge about hand hygiene (HH) and alcohol-based hand rub (ABHR) indications and HAIs. Unlike other instruments that were used in former related studies that utilized the concept of UPs in measuring knowledge about the present IPC practices [2, 13], the ICSQ is more specific in assessing the knowledge of HCWs about SPs and other IPC practices [12]. Additionally, the ICSQ has been used in several studies, including developed [14–17] and developing countries [18–20], given its international applicability because of its original English language form and its global relevance. However, to our knowledge, neither study provided any psychometric properties beyond Cronbach’s alpha.

In Hungary, two recent studies have employed a Hungarian version of the ICSQ (ICSQ-H) to assess IPC knowledge among nurses [21, 22]. However, only internal consistency was reported as a measure of psychometric properties. It is important, therefore, to establish a validated Hungarian version of the tool to facilitate a more comprehensive and precise measurement of knowledge about IPC among nurses in Hungary, given that Hungarian is the official language in Hungary. Furthermore, this tool may act as a basis for planning and performing interventions to enhance IPC knowledge. It will also ease more research concerning IPC knowledge to be conducted in Hungary, especially because, to our knowledge, there are no validated Hungarian tools to assess HCWs’ knowledge about IPC practices. Thus, the aim of this study was to assess the validity and reliability of the ICSQ-H in Hungarian nurses.

## Methods

### Study design and setting

This was a cross-sectional, multisite study. Seven hospitals from three counties of the southern Transdanubian region (Baranya, Somogy, and Tolna) of Hungary were included in this study.

### The questionnaire

The study used the ICSQ developed by Tavolacci et al. [12]. Approval for using the questionnaire was granted by Cambridge University Press. The questionnaire included two parts. The first part was meant to collect demographic information of the study participants, including age, gender, hospital, county, nursing department, educational degree, and years of experience. The second part involved 25 true/false questions regarding nurses’ awareness of three IPC topics: HAIs (five questions), HH (eight questions), and SPs (12 questions). The response to each question was coded and counted as not aware (0) and aware (1), where a maximum score of 25 was achievable for those who answered 25 correct questions. Additionally, an acceptable awareness score was set at 70% for each IPC topic as well as the total IPC awareness as per the original questionnaire [12].

### Translation

The translation of the ICSQ was performed following the recommended guidelines of translation, adaptation and validation of instruments for use in cross-cultural healthcare research [23]. The ICSQ was independently translated by two bilingual Hungarian nationals. Both were Ph.D. candidates in the health sciences and experts in the healthcare domain. The two Hungarian translated versions were reviewed and combined to produce a single version. This step was performed by a committee approach. Then, the synthesized Hungarian version was independently back-translated to English by two other bilingual Ph.D. candidates. Afterward, the two back-translated English versions were assessed by two individuals who synthesized them to produce a single back-translated Hungarian version. The first was a physician, while the second was a linguistic associate professor. Both had good knowledge of health terminology and IPC. First, the two back-translated versions were compared, and then they were compared against the original English version. This comparison was meant to assess similarities of the instrument questions, their wording, structure, meaning, and appropriateness.

### Content validity

The original ICSQ and the ICSQ-H were presented to a panel expert consisting of four members. The panel included an IPC specialist, a physician, and two nurses. The panel assessed the content validity of the ICSQ-H. Content validity determines the content representativeness or content relevance of the items of the studied instrument [24]. Content validity was established by calculating the item content validity index (I-CVI) and scale content validity index (S-CVI/Ave) [25]. As per Davis [26], a 4-point scale was used to rate the relevance of each item as follows: 1 = not relevant, 2 = somewhat relevant, 3 = quite relevant, and 4 = highly relevant. Then, for each item, the I-CVI was calculated as the number of experts giving a rating of either three or four divided by the total number of experts. The S-CVI/Ave was calculated as the average of I-CVIs by summing them and dividing by the number of items [25]. An I-CVI = 1 for a panel with ≤ 5 members [24] and an S-CVI/Ave ≥ 0.90 were acceptable [25]. After that, a pilot study was performed among 15 nurses. The nurses were asked to respond to the questionnaire and provide their comments on any items that they had difficulty understanding. None reported language problems or difficulty in answering the questions.

### Sample size

In general, it is recommended to use a minimum of 10 participants per item of the instrument scale in the case of exploratory factor analysis (EFA), which is equivalent to 250 participants in our case. However, in the case of EFA and confirmatory factor analysis (CFA), the recommendation is approximately 300–500 participants [23]. Based on this, we decided to include at least 500 nurses. Therefore, 810 questionnaires were distributed since we expected a low response rate due to the coronavirus disease (COVID-19) pandemic.

### Participants and data collection

Inclusion criteria for participation in this study included nurses who were working in inpatient units, including internal medicine, infectious diseases, surgery, critical care units, obstetrics-gynecology, hematology, oncology, and pediatrics, and who were willing to complete the questionnaire. To reduce nonresponse bias, hard copies of the questionnaires were distributed instead of online questionnaires. The head nurse of each unit administered the questionnaires to a convenience sample of nurses who were on schedule throughout the data collection period. Three months later, the completed questionnaires were collected by the researcher. Data collection was initiated in February 2020 and completed in May 2021.

### Statistical analysis

The Shapiro–Wilk test was used to check the normal distribution of the data. Frequencies as well as means and standard deviations (SD) were used to summarize the demographics of the participants. To manage missing data, incomplete questionnaires were disregarded. The structural validity of the ICSQ-H was assessed using principal component analysis (PCA) and CFA in a two-step process. Structural validity is the extent to which the scores of the instrument adequately reflect the dimensionality of the construct being measured [27]. Taking into consideration the recommendation of splitting the sample in construct-cross validation [28], we used a sample of 355 nurses who had more than 10 years of experience at their current hospital for the PCA. For the CFA, a sample of 236 nurses who had less than 10 years of experience was used.

In step one, SPSS was used. The Kaiser–Meyer–Olkin (KMO) was calculated to confirm the suitability of the data used for PCA (a value > 0.5 was acceptable), as well as a significant Bartlett’s test of sphericity (*p*-value < 0.05) [29]. For the extraction of factors, PCA was used, and Varimax with Kaiser Normalization was used as a rotation method in addition to an eigenvalue above one [30]. The rotated component matrix, scree plot, and parallel analysis were used to confirm the accurate number of factors to be retained [29].

In step two, a confirmative approach was adopted to validate the factor structure using the AMOS-23 program. Both the original model of the ICSQ and the PCA-suggested model were applied. Structural equation models in the CFA were evaluated by the overall goodness of fit for the models and by the value and significance of each parameter in the model. The goodness of fit for the model was evaluated through the following indices: the goodness-of-fit index (GFI > 0.95 well fit), the comparative fit index (CFI > 0.95 good fit), the Tucker-Lewis index (TLI > 0.95 good fit), the root mean square error of approximation (RMSEA < 0.06 good fit), the standardized root mean square residual (SRMR < 0.05 well fit), and the chi-square (χ2/df ratio < 3) with an insignificant *p*-value (> 0.05) [31].

Convergent and discriminant validity were evaluated using the Fornell and Larcker criterion [32]. Convergent validity indicates the level of correlation of multiple items of the same factor that are in agreement. This was met when the average variance extracted (AVE) value was above 0.5. Discriminant validity refers to the degree to which the factors are actually different from each other and do not measure the same thing. It was evaluated by calculating the Spearman correlation coefficient between the factors. A value of *r* < 0.3 indicated discriminant validity [33]. Additionally, discriminant validity was met when the square root of the AVE had a greater value than the correlations with other latent factors [32, 34].

The interitem correlations and the corrected item-total correlations were calculated. The interitem correlation shows the degree to which the items of the scales were related within the scales. A correlation between 0.2 and 0.85 was considered to indicate good consistency [35]. Correlations above 0.85 were considered redundant. Corrected item-total correlations are correlations between the scores from that question and the average scores of the other questions. A value ≥ 0.3 was considered acceptable [35]. Additionally, the internal consistency was evaluated using the Kuder-Richardson 20 (KR-20) coefficient. A value > 0.6 was considered sufficient [36].

### Ethical considerations

This study was approved by the Regional Research Ethics Committee of the Medical Center, Pécs, Hungary (Record number: 7862—PTE 2019). Before distributing the questionnaires, nurses were informed that their participation was voluntary and anonymous. All nurses signed written, informed consent forms. This study was reported as per the COSMIN reporting guideline for studies on measurement properties of patient‑reported outcome measures [37].

## Results

### Demographic characteristics

Of the 810 distributed questionnaires, 622 were returned, resulting in a response rate of 76.8%. Of them, 31 questionnaires were excluded due to missing data. Therefore, data from 591 nurses were analyzed. The mean age (± SD) of the participants was 41.93 ± 10.262. Nurses with more than 10 years of experience composed 60.1% of the sample. Out of all nurses, 91% were females, and 16.8% had a university nursing degree. The detailed demographics of the participants of both the PCA and CFA samples are shown in Table 1.

**Table 1:** Demographic characteristics of nurses

Demographic	Total sample *N* = 591	PCA sample *N* = 355	CFA sample *N* = 236
	**n (%)**	**n (%)**	**n (%)**
**Gender**			
Female	538 (91)	335 (94.4)	203 (86)
Male	53 (9)	20 (5.6)	33 (14)
**Hospital type**			
University	90 (15.2)	52 (14.6)	38 (16.1)
County	308 (52.1)	183 (51.5)	125 (53)
City	193 (32.7)	120 (33.8)	73 (30.9)
**County**			
Baranya	209 (35.4)	118 (33.2)	91 (38.6)
Tolna	204 (34.5)	144 (40.6)	60 (25.4)
Somogy	178 (30.1)	93 (26.2)	85 (36.0)
**Department**			
Medicine	137 (23.2)	86 (24.2)	51 (21.6)
Infectious	78 (13.2)	40 (11.3)	38 (16.1)
Surgery	104 (17.6)	60 (16.9)	44 (18.6)
Critical Care Units	89 (15.1)	51 (14.4)	38 (16.1)
Obstetrics-Gynecology	70 (11.8)	39 (11)	31 (13.1)
Hematology-Oncology	61 (10.3)	39 (11)	22 (9.3)
Pediatrics	52 (8.8)	40 (11.3)	12 (5.1)
**Educational degrees**			
University nursing degree	99 (16.8)	70 (19.7)	29 (12.3)
Vocational nursing training (OKJ)	383 (64.8)	226 (63.7)	157 (66.5)
Secondary school	109 (18.4)	59 (16.6)	50 (21.2)
**Age**	**Mean ± SD**	**Mean ± SD**	**Mean ± SD**
	41.93 ± 10.262	46.63 ± 7.425	34.86 ± 9.893

*PCA* Principal component analysis, *CFA* Confirmatory factor analysis, *SD* Standard deviation

### Content validity

After calculating the I-CVIs for each item in the ICSQ (25 items), two questions (Q 1D and 1E) had I-CVIs < 1. Therefore, both items were deleted. All other items had an I-CVI = 1. The S-CVI/Ave of the remaining 23 questions resulted in 1. Thus, our final questionnaire included 23 questions. Table 2 presents the detailed calculations of the I-CVI and S-CVI/Ave.

**Table 2:** Computation of the I-CVI and S-CVI/Ave with four expert raters

Items	**Expert 1** Infection prevention and control specialist	**Expert 2** Physician	**Expert 3** Nurse	**Expert 4** Nurse	Number in agreement of relevance	I-CVI
Q 1A	X	X	X	X	4	1
Q 1B	X	X	X	X	4	1
Q 1C	X	X	X	X	4	1
Q 1D	-	-	X	-	1	0.25*
Q 1E	-	-	X	-	1	0.25*
Q 2A	X	X	X	X	4	1
Q 2B	X	X	X	X	4	1
Q 2C	X	X	X	X	4	1
Q 2D	X	X	X	X	4	1
Q 3A	X	X	X	X	4	1
Q 3B	X	X	X	X	4	1
Q 3C	X	X	X	X	4	1
Q 3D	X	X	X	X	4	1
Q 4A	X	X	X	X	4	1
Q 4B	X	X	X	X	4	1
Q 4C	X	X	X	X	4	1
Q 4D	X	X	X	X	4	1
Q 5A	X	X	X	X	4	1
Q 5B	X	X	X	X	4	1
Q 5C	X	X	X	X	4	1
Q 5D	X	X	X	X	4	1
Q 6A	X	X	X	X	4	1
Q 6B	X	X	X	X	4	1
Q 6C	X	X	X	X	4	1
Q 6D	X	X	X	X	4	1
S-CVI/Ave (after deleting Q 1D and 1E)						1

*I-CVI* Item content validity index, *S-CVI/Ave* Scale content validity index average

- Ratings of 1 = not relevant, 2 = somewhat relevant. X Ratings of 3 = quite relevant, 4 = highly relevant. *I-CVI < 1 (item was deleted)

### Structural validity

The suitability for PCA was confirmed with a KMO measure of sampling adequacy of 0.650 and a significant Bartlett’s test of sphericity (χ2 = 2565.992; *p* < 0.001). PCA was performed on the ICSQ with 23 items. Six-factor solutions with eigenvalues greater than one were identified. The rotated component matrix, scree plot, and parallel analysis confirmed the six components, which accounted for a cumulative variance of 53.74%. Two items that failed to load at < 0.4 were removed (Q 1B and Q 6C). Q 3C was also removed since it loaded on two factors (one and two). Thus, 20 items remained. PCA was performed again (KMO = 0.646, Bartlett’s test of sphericity; χ2 = 2132.291; *p* < 0.001). Again, six-factor solutions with eigenvalues greater than one were identified and then confirmed, which accounted for a cumulative variance of 57.63%. After the second PCA run, another item was removed (Q 3B) due to loading on two factors (one and five). Furthermore, PCA was performed for the third time (KMO = 0.653, Bartlett’s test of sphericity (χ2 = 1944.372; *p* < 0.001). Five-factor solutions with eigenvalues greater than one were confirmed, which accounted for a cumulative variance of 52.80%. Two items that failed to load at < 0.4 were deleted again (Q 3A and Q 5D). Finally, PCA was performed for the last time (KMO = 0.686, Bartlett’s test of sphericity (χ2 = 1763.187; *p* < 0.001). Five-factor solutions with eigenvalues greater than one were identified and then confirmed, which accounted for a cumulative variance of 57.59%. An additional file shows the three PCA trials with loadings of all items (see Additional file 1).

Therefore, six items were deleted from the ICSQ at this stage. The remaining 17 items loaded on the following five factors: 1) use of gloves (GLVS) and SPs, 2) use of PPE, 3) ABHR indications on unsoiled hands, 4) SPs, and 5) HAIs and SPs, which are presented in Table 3. As shown in Table 3, the first factor included four items regarding the use of gloves and one item (Q 2C) with the lowest loading (0.419) on SPs, for that the factor was named “use of gloves (GLVS) and SPs”. Similarly, factor five included two items on HAIs and one (Q 4A) with the lowest loading (0.536) on SPs; thus, the factor was named “HAIs and SPs”.

**Table 3:** Principal component analysis of the infection control standardized questionnaire (*N* = 355)

Component	Item Nb	Item	**Component**				
			1	2	3	4	5
Use of gloves (GLVS) and Standard precautions (SPs)	Q 4B	The standard precautions recommend the use of gloves: When there is a risk of contact with the blood or body fluid	0.875				
	Q 4D	The standard precautions recommend the use of gloves: When healthcare workers have a cutaneous lesion	0.869				
	Q 4C	The standard precautions recommend the use of gloves: When there is a risk of a cut	0.680				
	Q 3D	Hand hygiene is recommended: After the removal of gloves	0.670				
	Q 2C	Standard precautions: Apply to all patients	0.419				
Use of personal protective equipment (PPE)	Q 5C	When there is a risk of splashes or spray of blood and body fluids, the healthcare workers must wear: Only a gown		0.928			
	Q 5B	When there is a risk of splashes or spray of blood and body fluids, the healthcare workers must wear: Only eye protection		0.926			
	Q 5A	When there is a risk of splashes or spray of blood and body fluids, the healthcare workers must wear: Only mask		0.898			
Alcohol-based hand rub (ABHR) indications on unsoiled hands	Q 6D	The indications for the use of alcohol-based hand rub (on unsoiled hands) are: Traditional handwashing must be done before handwashing with an alcohol-based hand rub			0.783		
	Q 6B	The indications for the use of alcohol-based hand rub (on unsoiled hands) are: Instead of antiseptic handwashing (30 s)			0.682		
	Q 6A	The indications for the use of alcohol-based hand rub (on unsoiled hands) are: Instead of traditional handwashing (30 s)			0.681		
SPs	Q 2A	Standard precautions: Include the recommendations to protect only the patients				0.747	
	Q 2D	Standard precautions: Apply for only healthcare workers who have contact with body fluids				0.742	
	Q 2B	Standard precautions: Include the recommendations to protect the patients and the healthcare workers				0.563	
Healthcare-associated infections (HAIs) and SPs	Q 1A	The environment (air, water, inert surfaces) is the major source of bacteria responsible for nosocomial infection					0.730
	Q 1C	Invasive procedures increase the risk of nosocomial infection					0.539
	Q 4A	The standard precautions recommend the use of gloves: For each procedure					0.536
Eigenvalues			2.905	2.704	1.578	1.401	1.202
Percentage of variance			17.089	15.904	9.283	8.243	7.070

**Fig. 1:**
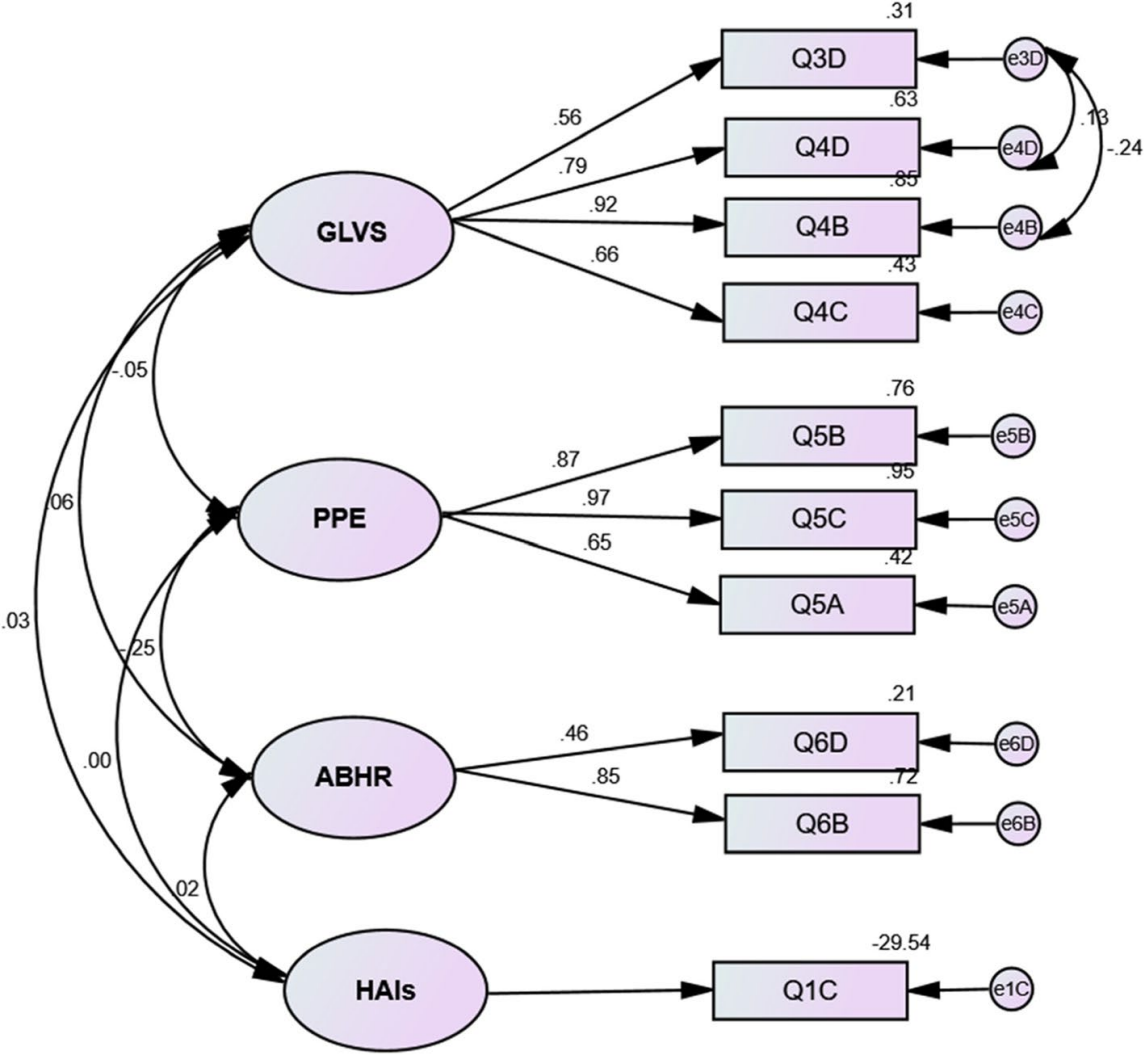
Confirmatory factor analysis of the four-factor model of the infection control standardized questionnaire Hungarian version. GLVS, use of gloves; PPE, use of personal protective equipment; ABHR, alcohol-based hand rub indications on unsoiled hands; HAIs, healthcare-associated infections

CFA was conducted using maximum likelihood. We evaluated the goodness of fit model by means of fit indices using AMOS software. First, the original structure of the ICSQ (23 items) was tested by CFA and resulted in a poor fit model with the following fit indices: χ2/df = 10.125;* p* < 0.001, GFI = 0.740, CFI = 0.487, TLI = 0.425, RMSEA = 0.124, SRMR = 0.1334. Therefore, our findings failed to support the original structure of the ICSQ. As a second step, our five-factor model identified by PCA was tested, which showed much-improved fit indices. However, this five-factor model showed a poor model fit (χ2/df = 2.007; *p* < 0.001, GFI = 0.9, CFI = 0.887, TLI = 0.860, RMSEA = 0.068, SRMR = 0.0733). Afterward, we deleted seven items due to low loadings (< 0.4). Q 1A and Q 4A were deleted from the “HAIs and SP” factor; thus, the factor was renamed HAIs. Additionally, Q 2C was removed from the “GLVS and SP” factor, so the factor was renamed GLVS. Furthermore, Q 6A was removed from the ABHR factor. Finally, the SP factor was deleted, including its three items Q 2A, Q 2B, and Q 2D. The new four-factor model including 10 items was tested again. The model showed a good fit, as all the indices indicated (χ2/df = 1.183; *p* = 0.231, GFI = 0.972, CFI = 0.994, TLI = 0.990, RMSEA = 0.028, SRMR = 0.0315). The standardized factor loadings of the items ranged from 0.46 to 0.97. The final four-factor model with the item loadings is shown in Fig. 1.

### Convergent and discriminant validity

Convergent validity was met except for the ABHR factor, which had an AVE value of 0.467, which is slightly less than 0.5. Discriminant validity was met for all factors since the square roots of the AVE were higher than the off-diagonal correlations between factors, as shown in Table 4. Additionally, weak correlations (*r* < 0.3) were found between the four factors.

**Table 4:** Convergent and discriminant validity of the four-factor infection control standardized questionnaire Hungarian version

**Factor**	**AVE**	GLVS	PPE	ABHR	HAIs
GLVS	0.555	**0.745**			
PPE	0.712	0.005	**0.844**		
ABHR	0.467	0.037	-0.181^b^	**0.683**	
HAIs	-	0.073	-0.024	0.141^a^	**-**

*AVE* Average variance extracted, *GLVS* use of gloves, *PPE* use of personal protective equipment, *ABHR* Alcohol-based hand rub indications on unsoiled hands, *HAIs* Healthcare-associated infections

^a^Correlation is significant at the 0.05 level (2-tailed)

^b^Correlation is significant at the 0.01 level (2-tailed)

### Internal consistency reliability

As shown in Table 5, the interitem correlations and the corrected item-total correlations of all factors were acceptable. The internal consistency was satisfactory for the GLVS and PPE factors, with KR-20 values of 0.780 and 0.897, respectively. The ABHR factor had a KR-20 of 0.529.

**Table 5:** Internal consistency reliability of factors

**Factor**	Interitem correlation	Corrected item-total correlation	Nb of items	Kuder-Richardson 20
GLVS	0.309–0.756	0.479–0.722	4	0.780
PPE	0.681–0.844	0.721–0.845	3	0.897
ABHR	0.360	0.360	2	0.529
HAIs	-	-	1	-

*GLVS* Use of gloves, *PPE* use of personal protective equipment, *ABHR* Alcohol-based hand rub indications on unsoiled hands, *HAIs* Healthcare-associated infections

## Discussion

This study aimed to evaluate the validity and reliability of the ICSQ-H. After translating the questionnaire to Hungarian, content validity was attained after removing two items. Then, the structural validity of the tool including 23 items was assessed using PCA and CFA. The final results of the PCA suggested a five-factor model with 17 items. Afterward, the CFA confirmed a four-factor model with 10 items. The original structure of the ICSQ (23 items) and the five-factor model suggested by the PCA did not meet the goodness of fit model requirements when tested for CFA. However, the final four-factor model (10 items) showed a good model fit where all the fit indices passed the requirements. The four factors of our suggested model were GLVS, PPE, ABHR, and HAIs. Furthermore, the convergent and discriminate validity of the instrument were tested and met for all factors except for the ABHR factor, where the convergent validity was slightly below the acceptable level. Additionally, the internal consistency of the factors was acceptable except for the ABHR factor.

Our findings did not support the original three-factor structure of the ICSQ. However, it should be clarified that the three factors (HAIs, HH, and SPs) that the original ICSQ evaluates are measured in our proposed Hungarian model (ICSQ-H) but with fewer items. For instance, the SP factor, including 12 items in the original ICSQ, was grouped into two factors in our model: the use of PPE and the use of GLVS, which measure the same parameter in the original questionnaire but with fewer items. Similarly, the HH factor, including eight items in the original ICSQ, can be found in our model as ABHR indications with two items, while another HH question was grouped with the GLVS factor, as it states the application of HH after removing gloves. Finally, in the original ICSQ, the HAI factor included five questions, while in our suggested model, it had only one item. We believe that failing to support the original structure of the ICSQ in our study could be due to the cultural and language differences between the French and Hungarian populations of nurses, as well as the difference in the policies and guidelines applied in the hospitals of the two countries, in addition to the differences in the educational systems and the curricula of nursing degrees that might affect the level of nurses’ IPC knowledge.

Our χ2/df was less than three with an insignificant *p*-value, which indicates a good model fit. However, there are some limitations for χ2/df model use. The main limitation is having a small sample size where χ2/df lacks power and might not be able to distinguish between good fitting models and poor fitting models [31, 38]. When having a large sample size, the χ2/df model is exact, which is our case [38]. Our results showed that GFI, CFI, and TLI values were above 0.95. Given the detrimental effect of the sample size on the GFI index, it is recommended to be used along with other indices that we took into account when conducting our study [31]. For instance, CFI is one of the most used and recommended fit indices since it is among the measures least affected by sample size. Similarly, TLI is a fit index that is less affected by sample size. In this study, the values of both CFI and TLI indicated a good model fit [31]. RMSEA has recently been suggested as one of the most informative fit indices since it is affected by the total count of the estimated parameters in the model. Until the early 1990s, a value between 0.05 and 1 was considered to reflect a fair model fit [31, 39]; however, in the late 1990s, a value less than 0.06 was recommended [31, 40]. Our model showed a much lower RMSEA, which indicates the goodness of fit of the model. Additionally, SRMR is recommended for use since it is easier to interpret than other fit indices because of its standardized nature. Values closer to zero show a better fit, which is the case for our model [31].

Convergent validity was met for the GLVS and PPE factors, which indicates a satisfactory level of correlation of multiple items of the same factor [34]. However, the AVE of the ABHR factor was slightly below 0.5, which could still be considered acceptable. The weak correlations between the four factors proved the discriminant validity of each. This means that the measures of distinct factors share a little common variance and support the uniqueness of the items and the factor [33]. Furthermore, it indicates that the latent factors used for measuring the causal relationships in our model are actually different from each other and do not measure the same thing that could lead to multicollinearity [34].

Concerning the interitem correlations and the corrected item-total correlations, they were acceptable for all factors. Furthermore, the internal consistency of the ABHR factor was below 0.6; however, its interitem correlations and the corrected item-total correlations were acceptable. This could be due to the low number of items in this factor (two items) [41].

Finally, the removal of 15 items during the different stages of this study (two items during content validity assessment, six items during PCA, and seven items during CFA) might considerably modify the original factor structure of the ICSQ, bearing in mind that they could hold valuable and important factors in IPC. Nevertheless, these findings further suggest the existence of repetitions of similar items measuring similar factors that compromise the construct validity of the original ICSQ [42]. However, the concise methodology that we have used allows for an adequate start to develop a Hungarian tool to assess IPC knowledge among the Hungarian population.

Few studies have been conducted to test the psychometric properties of some IPC questionnaires that are used to assess HCWs’ knowledge about IPC measures. For instance, Duarte Valim et al. [43] validated the Knowledge Questionnaire regarding Standard Precautions Measures (QCSP) for Brazilian nurses. Convergent validity was tested using known-group methods. Reliability was tested by calculating the intraclass correlation coefficient (ICC) by applying the test–retest method. The Kappa index was used for the purpose of agreement. The Portuguese QCSP showed satisfactory ICC and Kappa. However, validation by discriminant groups did not reveal a statistically significant difference between the two groups. Similarly, the infection control evaluation tool was developed by Wu et al. [2] to assess nursing students’ knowledge about standard and additional IPC precautions. The tool was a modified version derived from two previously developed tools including 15 questions. Content validity was assessed by six experts using the CVI, where an acceptable degree of validity was found, with 68% agreement. KR-20 was used to test the internal consistency, which revealed a satisfactory value of 0.76. It is worth mentioning that this tool was based on two previously developed tools, mainly Chan et al. [13], who employed the concept of UPs in measuring knowledge. Another tool was developed by Chan et al. [44] in 2008 to examine nurses’ knowledge of SPs and transmission-based precautions using four multiple-choice questions. Content validity was assessed by two experts with a CVI = 0.97. Structural validity was assessed using EFA. One factor was found to include four items with factor loadings ranging from 0.76 to 0.86. The scale reliability was assessed via test–retest. Cronbach’s alpha showed an acceptable value (0.79). Finally, we noticed that only one study assessed the structural validity of the scale using EFA [44], while neither study performed CFA, which suggests that further research is needed to test the structural validity of these scales using EFA and CFA.

### Strengths and limitations

Our study is the first to test the psychometric properties of the ICSQ-H. Although the study was performed in the southern Transdanubian region of Hungary, we included all hospital types (university, county, and city) from different counties, so we believe that our results could be generalized to reflect the situation across Hungary. However, our study has some limitations. First, using convenience sampling might have introduced selection bias. Second, two factors in our model include fewer than three items. Generally, models containing more items per factor are preferred since they show more accurate parameter estimates and greater reliability. Nevertheless, the ICSQ-H could act as the first step in conducting more research on the development of Hungarian tools that assess nurses’ IPC knowledge. Another limitation is that we could not compare our results to other existing models. Although the ICSQ has been used in several countries to assess HCWs’ knowledge about IPC, its psychometric properties have not been tested and reported in other languages. Thus, future studies are needed to test the psychometric properties of the ICSQ in other languages and settings. Finally, our data were collected during the COVID-19 pandemic, so we are uncertain if the awareness level of nurses was affected due to their high alertness during this period.

### Relevance to practice and research

Given that Hungarian is the official language in Hungary, it was necessary to validate a Hungarian tool to facilitate a more comprehensive and precise measurement of knowledge about IPC among nurses in Hungary. Based on our findings, we believe that the ICSQ-H could pave the way for more research regarding nurses’ IPC knowledge to be conducted in Hungary. Additionally, several studies have shown that the length of the instrument has a negative relation with the participants’ response rate [45]. Due to the time limits of nurses, especially currently during the COVID-19 pandemic, our ICSQ-H was found to be short and feasible. Nevertheless, its validation among other HCWs is important to tailor effective interventions to enhance knowledge and awareness. On the other hand, our model includes two factors with less than three items, which is not optimal; however, these findings might be a start to think about having more research regarding developing a Hungarian tool to assess IPC knowledge among Hungarian nurses.

## Conclusion

This study did not support the original three-factor structure of the ICSQ tool. However, the ICSQ-H based on the four-factor structure revealed by PCA and CFA demonstrated an adequate degree of good fit and was found to be reliable. The ICSQ-H could contribute to conducting more research on the development of Hungarian tools that assess nurses’ IPC knowledge among the Hungarian population. Further research is needed to test the psychometric properties of the ICSQ across different countries and languages.

## Supplementary Information

**Additional file 1.** Principal Component Analysis (PCA) trials of the Infection Control Standardized Questionnaire (ICSQ).

## Data Availability

The datasets generated and/or analyzed during the current study are not publicly available due [privacy, “data contain information that could compromise research participant privacy/consent”] but are available from the corresponding author on reasonable request.

## Abbreviations

IPC: Infection prevention and control
HAIs: Healthcare-associated infections
HCWs: Healthcare workers
Ups: Universal precautions
SPs: Standard precautions
ICSQ: Infection control standardized questionnaire
PPE: Personal protective equipment
HH: Hand hygiene
ABHR: Alcohol-based hand rub
ICSQ-H: Hungarian version of the ICSQ
I-CVI: Content validity index
S-CVI/Ave: Scale content validity index
EFA: Exploratory factor analysis
CFA: Confirmatory factor analysis
COVID-19: Coronavirus disease
SD: Standard deviations
PCA: Principal component analysis
KMO: The Kaiser–Meyer–Olkin
GFI: The Goodness-of-Fit index
CFI: The comparative fit index
TLI: The Tucker-Lewis index
RMSEA: The root mean square error of approximation
SRMR: The standardized root mean square residual
AVE: The average variance extracted
KR-20: Kuder-Richardson 20
GLVS: Gloves
QCSP: Knowledge Questionnaire regarding Standard Precautions Measures
ICC: The Intraclass correlation coefficient
