# Supplementary material for: Translation and validation of the Hungarian Version of the infection control standardized questionnaire: a cross-sectional study
Source: BMC Nurs. 2022 Sep 2;21:244. doi: 10.1186/s12912-022-01024-8 (PMC9436728; doi:10.1186/s12912-022-01024-8)
Supplement: Supplementary file 1 — Additional file 1. Principal Component Analysis (PCA) trials of the Infection Control Standardized Questionnaire (ICSQ). [file 12912_2022_1024_MOESM1_ESM.docx]

**Additional file 1.**

Title of data: Principal Component Analysis (PCA) trials of the Infection Control Standardized Questionnaire (ICSQ).

Description of data: The tables show the three PCA trials of the ICSQ with all item loadings.

PCA 1 of the ICSQ (N =355)

|  |  | **Component** | | | | | |
| --- | --- | --- | --- | --- | --- | --- | --- |
| Item |  | 1 | 2 | 3 | 4 | 5 | 6 |
| Q 4D | The standard precautions recommend the use of gloves: When healthcare workers have a cutaneous lesion. | 0.838 |  |  |  |  |  |
| Q 4B | The standard precautions recommend the use of gloves: When there is a risk of contact with the blood or body fluid. | 0.831 |  |  |  |  |  |
| Q 3D | Hand hygiene is recommended: after the removal of gloves. | 0.717 |  |  |  |  |  |
| Q 4C | The standard precautions recommend the use of gloves: When there is a risk of a cut. | 0.664 |  |  |  |  |  |
| Q 2C | Standard precautions: Apply to all patients. | 0.405 |  |  |  |  |  |
| Q 5B | When there is a risk of splashes or spray of blood and body fluids, the healthcare workers must wear: Only eye protection. |  | 0.918 |  |  |  |  |
| Q 5C | When there is a risk of splashes or spray of blood and body fluids, the healthcare workers must wear: Only a gown. |  | 0.878 |  |  |  |  |
| Q 5A | When there is a risk of splashes or spray of blood and body fluids, the healthcare workers must wear: Only mask. |  | 0.805 |  |  |  |  |
| Q 3B | Hand hygiene is recommended: Before and after a contact with (care of) a patient. |  | 0.686 |  |  |  |  |
| Q 6D | The indications for the use of alcohol-based hand rub (on unsoiled hands) are: Traditional handwashing must be done before handwashing with an alcohol-based hand rub. |  |  | 0.732 |  |  |  |
| Q 6B | The indications for the use of alcohol-based hand rub (on unsoiled hands) are: Instead of antiseptic handwashing (30 seconds). |  |  | 0.700 |  |  |  |
| Q 6A | The indications for the use of alcohol-based hand rub (on unsoiled hands) are: Instead of traditional handwashing (30 seconds). |  |  | 0.684 |  |  |  |
| Q 2A | Standard precautions: Include the recommendations to protect only the patients. |  |  |  | 0.741 |  |  |
| Q 2D | Standard precautions: Apply for only healthcare workers who have contact with body fluids. |  |  |  | 0.682 |  |  |
| Q 2B | Standard precautions: Include the recommendations to protect the patients and the healthcare workers. |  |  |  | 0.446 |  |  |
| Q 3A | Hand hygiene is recommended: Before or after a contact with (care of) a patient. |  |  |  |  | 0.636 |  |
| Q 5D | When there is a risk of splashes or spray of blood and body fluids, the healthcare workers must wear: Mask, goggles, and gowns. |  |  |  |  | 0.596 |  |
| Q 1A | The environment (air, water, inert surfaces) is the major source of bacteria responsible for nosocomial infection. |  |  |  |  |  | 0.636 |
| Q 4A | The standard precautions recommend the use of gloves: For each procedure. |  |  |  |  |  | 0.548 |
| Q 1C | Invasive procedures increase the risk of nosocomial infection. |  |  |  |  |  | 0.534 |
| Q 3C | Hand hygiene is recommended: Between patient contacts. | 0.636 | 0.423 |  |  |  |  |
| Q 1B | Advanced age or very young age increases the risk of nosocomial infection. |  |  |  |  |  |  |
| Q 6C | The indications for the use of alcohol-based hand rub (on unsoiled hands) are: Instead of surgical handwashing (3 minutes). |  |  |  |  |  |  |

Total number of items = 23

PCA 2 of the ICSQ (N =355)

|  | **Component** | | | | | |
| --- | --- | --- | --- | --- | --- | --- |
| Item | 1 | 2 | 3 | 4 | 5 | 6 |
| Q 5B | 0.929 |  |  |  |  |  |
| Q 5C | 0.889 |  |  |  |  |  |
| Q 5A | 0.827 |  |  |  |  |  |
| Q 3B | 0.675 |  |  |  | -0.431 |  |
| Q 4D |  | 0.866 |  |  |  |  |
| Q 4B |  | 0.861 |  |  |  |  |
| Q 4C |  | 0.688 |  |  |  |  |
| Q 3D |  | 0.672 |  |  |  |  |
| Q 2C |  | 0.419 |  |  |  |  |
| Q 6D |  |  | 0.777 |  |  |  |
| Q 6A |  |  | 0.712 |  |  |  |
| Q 6B |  |  | 0.679 |  |  |  |
| Q 2D |  |  |  | 0.747 |  |  |
| Q 2A |  |  |  | 0.738 |  |  |
| Q 2B |  |  |  | 0.530 |  |  |
| Q 5D |  |  |  |  | 0.659 |  |
| Q 3A |  |  |  |  | 0.633 |  |
| Q 1A |  |  |  |  |  | 0.696 |
| Q 4A |  |  |  |  |  | 0.545 |
| Q 1C |  |  |  |  |  | 0.544 |

Total number of items = 20

PCA 3 of the ICSQ (N =355)

|  | **Component** | | | | |
| --- | --- | --- | --- | --- | --- |
| Item | 1 | 2 | 3 | 4 | 5 |
| Q 4B | 0.866 |  |  |  |  |
| Q 4D | 0.865 |  |  |  |  |
| Q 4C | 0.685 |  |  |  |  |
| Q 3D | 0.677 |  |  |  |  |
| Q 2C | 0.414 |  |  |  |  |
| Q 5C |  | 0.926 |  |  |  |
| Q 5A |  | 0.895 |  |  |  |
| Q 5B |  | 0.877 |  |  |  |
| Q 6D |  |  | 0.776 |  |  |
| Q 6B |  |  | 0.681 |  |  |
| Q 6A |  |  | 0.657 |  |  |
| Q 2D |  |  |  | 0.773 |  |
| Q 2A |  |  |  | 0.728 |  |
| Q 2B |  |  |  | 0.499 |  |
| Q 1A |  |  |  |  | 0.678 |
| Q 1C |  |  |  |  | 0.551 |
| Q 4A |  |  |  |  | 0.538 |
| Q 5D |  |  |  |  |  |
| Q 3A |  |  |  |  |  |

Total number of items = 19
